# Towards propagation of epidermal cells for wound repair: glass, as cell culture substrate, enhances proliferation and migration of human keratinocytes

**DOI:** 10.3389/fbioe.2025.1547044

**Published:** 2025-03-20

**Authors:** Hady Shahin, Ingrid Steinvall, Folke Sjöberg, Moustafa Elmasry, Ahmed El-Serafi

**Affiliations:** ^1^ Department of Hand Surgery, Plastic Surgery and Burns, Linköping University, Linköping, Sweden; ^2^ The Department of Biomedical and Clinical Sciences, Linköping University, Linköping, Sweden; ^3^ Faculty of Biotechnology, Modern Sciences and Arts University, Cairo, Egypt

**Keywords:** keratinocytes, culture substrate, glass, epidermal differentiation, migration, ATMP, wound healing

## Abstract

**Introduction:**

Human keratinocytes require relatively long propagation time which impedes their availability as autologous cell transplantation within a clinically reasonable timeframe. There is an unmet need for efficient xeno-free cell expansion approaches to propagate human keratinocytes as regenerative therapy.

**Methods:**

Primary human keratinocytes and HaCaT cells were cultured on glass, plastic, and animal-derived collagen I matrix for 10 days. Proliferation, migration, DNA methylation, as well as gene and protein expression were assessed to characterize the effect of the tested culture substrates on keratinocytes at the molecular and functional levels.

**Results:**

Keratinocytes cultured on glass exhibited faster proliferation, global DNA demethylation and upregulation of epidermal differentiation markers. Scratch wound assay revealed that keratinocytes cultured on glass demonstrated enhanced cell migration compared to those on plastic or collagen I. Multiplex immunoassays identified temporal and substrate-dependent variations in a panel of keratinocyte-specific secreted factors, encompassing immunomodulatory cytokines, growth factors, and angiogenic factors.

**Discussion:**

Glass, as a culture substrate, promotes epidermal differentiation and enhances keratinocyte migration. The latter is a critical factor in re-epithelialization and wound healing. Functional properties suggest that glass may optimize the inflammatory response and promote efficient wound repair, making it a promising candidate for the short-term expansion of keratinocytes for transplantation purposes. Further *in-vivo* validation is required to definitively establish the efficacy of keratinocytes cultured on glass for clinical applications.

## 1 Introduction

Difficult-to-heal skin wounds remain as a considerable challenge in healthcare with debilitating socio-economic burdens (Jarbrink et al., 2017). The application of cultured autologous epithelial keratinocytes has been heavily investigated as a line of management for difficult-to-heal wounds, with reported advantageous outcomes including faster healing, better skin quality, reduced scar formation, in addition to overcoming the challenges associated with skin grafts (Fang et al., 2014; Gardien et al., 2016; Johnstone et al., 2017; Ter Horst et al., 2018; Karlsson et al., 2020). However, the procedure of primary keratinocyte expansion to reach satisfactory cell yield for transplantation can be time consuming, which does not meet the timeframe for clinical need. Delayed interference can lead to prolonged healing times and undesired complications (Ter Horst et al., 2018). Furthermore, long-term expansion of epidermal keratinocytes is often associated with progressive loss of proliferation capacity and cell characteristics along the passages (Esteban-Vives et al., 2015). Therefore, safe, effective and short-term expandability of keratinocytes are indispensable prerequisites for potential cell transplantation solutions in clinical applications.

The classical approach to routinely culture human keratinocytes was established by Rheinwald and Green in 1975. Regarded by many as the “gold standard” for human keratinocyte cultures, their procedure covers the entire process from enzymatic and mechanical dissociation of epidermis to maintaining serially cultivated keratinocytes in culture. However, since dermal fibroblasts removal from contact with the keratinocytes impedes their growth; an irradiated layer of mouse embryonic fibroblast/feeder cells were incorporated into the system (Rheinwald and Green, 1975). The need for feeder layer got overridden with the advent of advanced culture medium supplementations, extracellular matrix (ECM) substrates and coating materials. Studies have shown primary keratinocyte cultures successfully established on surfaces coated with gelatin (Daniels et al., 1997), recombinant laminins (Tjin et al., 2018), and type IV collagen. The latter showed gradual suppression of keratinocytes proliferation (Fujisaki et al., 2008). Alternatively, rat-tail type I collagen coating matrices have been broadly used to expand keratinocytes as they promote cell proliferation, attachment and growth (Horch et al., 2000; Lenihan et al., 2014; Esteban-Vives et al., 2015; Orazizadeh et al., 2015; Wong et al., 2019). The European Medicines Agency (EMA) has claimed the process of manufacturing and transplanting cultured cells under the directives for developing advanced therapy medicinal products (ATMP). This new regulatory framework necessitates the riddance of animal-derived products from the process of developing autologous cultured keratinocyte-transplantation solutions (Shahin et al., 2020; Lagerwall et al., 2021). We have demonstrated the possibility and efficacy of a xeno-free keratinocyte extraction workflow as well as the establishment of keratinocyte cultures in xeno-free condition. However, even the xeno-free media requires usually surface coating with collagen-based matrix, which is generally of animal origin (Lagerwall et al., 2021).

The choice of an appropriate xeno-free surface supporting keratinocytes colonization and expandability remains a missing link in our standard operating procedure for good manufacturing practice compliant keratinocyte-based transplantation solution. Historically, glass has been used as a substrate for cell culture as it supports cell attachment (Barker and Larocca, 1994). The physical features of borosilicate-based materials, including surface composition, topography and charge, contribute to their appeal. Bioactive glass has been instrumental in field of orthopedic therapeutics due to their osteoconductivty (Nugud et al., 2022). Several reports showed the effect of glass on enhancing angiogenesis and osteogenic differentiation of human mesenchymal stem cells (Curran et al., 2005; Nicholas et al., 2005; Westhauser et al., 2019; Alghfeli et al., 2021), and human umbilical cord perivascular cells (Kargozar et al., 2017). Nevertheless, to date, there has been a scarcity of evidence on the effect of glass on expanding epidermal cells and its impact on epidermal differentiation, in contrast to standard culture plastic ware and animal-derived ECM substrates.

Herein, we report a xeno-free and matrix-free culture substrate utilizing borosilicate-based glass in expanding monolayer of human keratinocytes. The efficacy of the glass substrate is compared with commercially available culture plastic and rat-tail collagen I coating. This study is an attempt to develop an EMA compliant expansion protocol which enables expeditious propagation of adult human keratinocytes *in-vitro* to facilitate their scalability for ATMP bio-production purposes.

## 2 Materials and methods

### 2.1 Primary cell isolation and cell lines

Split thickness skin biopsies were obtained from healthy donors during abdominoplasty and/or breast reduction procedures, under the ethical approval no. 2015/177–31 by the Swedish Ethical Review Authority. The skin was cut into 2–3 mm^2^ and incubated overnight in 1:1 volume of 10 mg/mL Dispase II solution (Life Technologies, Japan) at 4°C. The epidermis was then gently peeled from the dermis and incubated with Trypsin-EDTA (Sigma-Aldrich, United States) on a tube revolver at 37°C for 30 min. Trypsin was deactivated by media containing 10% fetal bovine serum (Life Technologies, Brazil). The cell suspension was allowed to pass through a 70 μm cell strainer (Corning, NY, United States), then the keratinocyte suspension was centrifuged at 2,900 × g for 4 min. The cell pellets were washed twice with phosphate buffered saline (PBS; Gibco, Life Technologies, United Kingdom) before cells were resuspended in keratinocyte serum free media (KSFM; Gibco, Life Technologies, United Kingdom) supplemented with bovine pituitary extract and epidermal growth factor (Thermo Fisher, New Zealand); in addition to 10,000 units penicillin and 10 mg streptomycin/mL (Sigma-Aldrich, United States). HaCaT (Elabscience Biotechnology Inc., United States), a cell line of spontaneously transformed keratinocytes isolated from histologically normal skin, was used to model epidermal keratinocytes in certain assays. HaCaT cell line was expanded in complete DMEM (Gibco, Life Technologies, United Kingdom) with 10% fetal bovine serum (Life Technologies, Brazil) and 10,000 units penicillin and 10 mg streptomycin/mL (Sigma-Aldrich, United States).

### 2.2 Culture condition on substrates

Upon reaching 90% confluence, cultured human keratinocytes cells were dissociated with Trypsin-EDTA (Sigma Aldrich, United States), stained with 0.4% trypan blue (1:1) and counted using a TC20 automated cell counter (Bio-Rad Inc., Singapore). Primary keratinocytes were seeded in monolayer at passage one onto plastic, cell culture-treated multi-well plates (Corning™, Costar™, United States), collagen I coated multi-well plates (Thermo Scientific™ Nunc™, United States) and glass bottom multidishes (Cellvis™, Sunnyvale, CA, United States) at a density of 2.5 × 10^4^ cell/cm^2^ in KSFM media. HaCaT were seeded on the same substrates in complete DMEM. Cells were cultured on the substrates for 10 days while allowing for downstream investigations at various time points in the interim.

### 2.3 Cell proliferation

HaCaT in four independent cell line replicates (n = 4) were seeded onto the different culture substrates in monolayer. Cells were trypsinized at 24, 48 and 72-hour time points, stained with 0.4% trypan blue (1:1) and counted using a TC20 automated cell counter (Bio-Rad Inc., Singapore). Viability percentage was recorded as well as total cell count, based on the following equation:

$$\text{Viability percentage}=\frac{\text{Total}\;\text{number}\;\text{of}\;\text{viable}\;\text{cells}\;\text{per}\;1\;\text{mL}\;}{\text{Total}\;\text{number}\;\text{of}\;\text{cell}\;\text{per}\;1\;\text{mL}\;}\;X\;100$$

The growth curve was calculated according to the following equation: Normalized cell count = C_t_/C_0_ (C_t_: viable cell count at time point of interest, C_0_ = viable cell count at initial seeding).

$$\text{Normalized cell count}=\frac{\text{Viable}\;\text{cell}\;\text{count}\;\text{at}\;\text{time}\;\text{point}\;\text{of}\;\text{interest}\;}{\text{Viable}\;\text{cell}\;\text{count}\;\text{at}\;\text{initial}\;\text{seeding}}$$

### 2.4 Gene expression analysis

Quantitative real-time PCR was performed to detect the expression of lineage specific, apoptotic and ECM genes from four independent replicates of HaCaT, cultured on the three substrates (n = 4) for 10 days. Total cellular RNA was extracted using RNeasy mini kit (Qiagen, Hilden, Germany). One µg RNA was reverse transcribed into cDNA using QuantiTect reverse transcription kit (Qiagen, Germany) as recommended by the manufacturer. The PowerUp^©^ SYBR green master mix (Applied biosystem, Waltham, MA, United States) was used in conjunction with the oligonucleotide primers for the target genes listed in Table 1. Gene expression levels were quantified in a 7,500 Fast Real-Time PCR System (Applied Biosystem, Thermo Fisher, United States) and performed in a minimum of three technical replicates. Expression of the genes of interest was normalized against the endogenous control Glyceraldehyde 3-phosphate Dehydrogenase (GAPDH) and the fold change was calculated with the 2^−ΔΔCT^ method (Livak and Schmittgen, 2001). The reference for each gene was its expression by cells cultured on plastic.

**TABLE 1:** Primer sequences of studied genes of interest.

Target	Forward primer	Reverse primer	References
GAPDH	CCT​GCA​CCA​CCA​ACT​GCT​TA	GGC​CAT​CCA​CAG​TCT​TCT​GAG	Hu et al. (2021)
p63	GAA​AAC​AAT​GCC​CAG​ACT​CAA	TGCGCGTGGTCTGTGTTA	Herfs et al. (2010)
K1	GTT​CCA​GCG​TGA​GGT​TTG​TT	TAA​GGC​TGG​GAC​AAA​TCG​AC	Alam et al. (2011)
K5	CAG​AGC​CAC​CTT​CTG​CGT​CCT​G	GCT​GAA​GCT​ACG​ACT​GCC​C	Chavez-Munoz et al. (2013)
K10	CCA​TCG​ATG​ACC​TTA​AAA​ATC​AG	GCA​GAG​CTA​CCT​CAT​TCT​CAT​ACT​T	Omori-Miyake et al. (2014)
K14	CCT​CCT​CCA​GCC​GCC​AAA​TCC	TTG​GTG​CGA​AGG​ACC​TGC​TCG	Chavez-Munoz et al. (2013)
Involucrin	TCC​TCC​AGT​CAA​TAC​CCA​TCA​G	CAG​CAG​TCA​TGT​GCT​TTT​CCT	Chavez-Munoz et al. (2013)
Stratifin	ACT​TTT​CCG​TCT​TCC​ACT​ACG​A	ACA​GTG​TCA​GGT​TGT​CTC​GC	Chavez-Munoz et al. (2013)
Filaggrin	TGA​AGC​CTA​TGA​CAC​CAC​TGA	TCC​CCT​ACG​CTT​TCT​TGT​CCT	Chavez-Munoz et al. (2013)
Loricrin	CAT​GAT​GCT​ACC​CGA​GGT​TT	AAC​CAA​AGA​GGC​TAA​ACA​GCA	Yang et al. (2010)
Col IV	GGA​CAG​ACG​AGA​CAA​CAG​CA	GAG​CTG​GCA​TAA​CAT​TGG​CG	Limandjaja et al. (2018)
Fibronectin	GAG​AAT​GGA​CCT​GCA​AGC​CCA	GTG​CAA​GTG​ATG​CGT​CCG​C	Park et al. (2018)
MMP1	ATT​CTA​CTG​ATA​TCG​GGG​CTT​TGA	ATG​TCC​TTG​GGG​TAT​CCG​TGT​AG	Tang et al. (2019)

Abbreviations: GAPDH, Glyceraldehyde 3-phosphate Dehydrogenase; K1, 5, 10 & 14, Keratin 1, 5, 10 & 14; Col IV, Collagen IV; MMP1, Matrix metalloproteinase-1.

### 2.5 Thrombospondin-1 quantification

Culture supernatant samples were collected at days 7 and 10, from 3 independent cell line replicates cultured on the three substrates (n = 3). Human Thrombospondin-1 ELISA kit (Abcam, Cambridge, United Kingdom) was used as instructed in the manufacturer’s manual to quantify the amount of THBS1 released by the cells in response to their corresponding culture substrate. Briefly, standards and samples were incubated for 2.5 h at room temperature to allow the immobilized antibody in the plate wells to capture thrombospondin-1 present in the samples. The wells were washed and biotinylated Human thrombospondin-1 antibody was added for 1 h at room temperature. Then, an HRP-conjugated streptavidin was added and incubated for 45 min at room temperature. After incubation, the wells were washed, followed by the addition of a one-step substrate solution to the wells. The intensity of the developed colour was read at 450 nm using SpectraMax Plus 384 microplate reader (Molecular Devices, United States). The quantitative measurement of THBS1 was calculated using a standard curve with a coefficient of determination of R^2^ = 0.9574. The measured absorbance values were proportionated to the concentration of THBS1 (ng/mL) released in the culture supernatants and bound to the assay wells.

### 2.6 Global DNA methylation analysis

Genomic DNA was extracted from three independent replicates of primary keratinocytes cultured on the three substrates (n = 3) for 10 days, using RNA/DNA/Protein purification plus kit (Norgen Biotek, Canada) according to manufacturer’s instructions. Briefly, the cell lysate was loaded into the genomic DNA (gDNA) isolation column and the eluted gDNA was quantified using the NanoDrop 1,000 spectrophotometer (Thermo Fisher Scientific, Waltham, MA, United States). DNA Methylation Quantification Kit (Abcam, Cambridge, United Kingdom) was used to quantify the amount of methylated DNA colorimetrically following the steps in the manufacturer’s manual. Briefly, 100 ng of DNA were bound to the assay wells according to the manufacturer instructions by incubating with binding solution for 90 min at 37°C. Then the capturing antibody for 5-methylcytosine (5-mC) was added to each well and incubated for 60 min at room temperature. Finally, the detection antibody followed by enhancer and developer solution were added. The absorbance of the developed colour was read spectrophotometrically at 450 nm using SpectraMax Plus 384 microplate reader (Molecular Devices, United States). Global 5-mC (ng) was calculated using a standard curve showing a coefficient of determination of R^2^ = 0.9986.

### 2.7 Cell migration

Cell migration was determined by the scratch wound assay (SWA) on the three culture substrates. Primary keratinocytes from three independent donors (n = 3) were seeded at passage 1 into 6-well plates at a density of 2.5 × 10^4^ cell/cm^2^ in KSFM media. Cells were allowed to grow until they formed a semi-confluent monolayer, then a straight vertical scratch was made down through the centre of the well by using a sterile 100 µL plastic pipette tip, followed by a gentle wash and media change. The gap was imaged under inverted microscope equipped with a digital camera (CKX53, Olympus Corp., Tokyo, Japan) using the Imageview software version X64 (Olympus Corp., Japan) at 0, 6, 12, 18 and 24 h. The area of the gap was measured using Photoshop version 24.3.0 (Adobe Inc. San Jose, CA, United States). The cellular migration rate was calculated according to the following equation:

$$\text{Wound closure }\left(\%\right)=\left[\frac{At\left(0h\right)-At\left(∆h\right)}{At\left(0h\right)}\right]x\;100\%$$

Where, A_t(0h)_ = is the area of the wound measured immediately after scratching (time zero), and A_t(∆h)_ is the area of the wound measured at h hours after the scratch is performed (Martinotti and Ranzato, 2020).

### 2.8 Luminex^®^ multiplex immunoassays

The culture supernatant was collected from four independent HaCaT replicates cultured on the three different substrates (n = 4) at days 3, 7, and 10. Total protein concentration was assayed using the DC protein assay (Bio-Rad Inc., Hercules, CA, United States) following the manufacturer’s instructions. Luminex^®^ bead-based assays (R&D Systems, Inc., Minneapolis, MN, United States) were employed for the simultaneous detection and quantification of multiple analytes released by the cultured cells in response to their respective culture substrates. The premixed human analytes assayed were Interleukin one alpha (IL-1α), Interleukin 8 (IL-8), Epidermal Growth Factor (EGF), Tissue Inhibitor of Metalloproteinases 1 (TIMP-1), Tumor Necrosis Factor (TNF-α), Vascular Endothelial Growth Factor A (VEGF-A), and Kallikrein-Related Peptidases 5 and 6 (KLK5 & KLK6).

Following the manufacturer’s instructions, 50 µL of the standard cocktail and culture supernatant samples were added to their designated wells in the microplate. The bead mixture, together with the assay buffer, was diluted and 50 µL of the microsphere bead cocktail was added to each well. The plate was incubated for 2 h on an orbital shaker at room temperature, allowing the analytes in the samples and standards to bind to the specific antibodies on the beads. The plate was then washed using a magnetic plate washer by applying the magnet to the bottom of the microplate to ensure uniform removal of liquids and unbound substances. A biotinylated secondary antibody cocktail was added and incubated for 1 h at room temperature to bind to the captured analytes on the beads. After washing off the unbound secondary antibody, Streptavidin-phycoerythrin (SA-PE) was added for detection and incubated for 30 min on an orbital shaker. Finally, the microsphere beads were resuspended on a plate shaker for 2 min at 800 rpm immediately prior to detection. The Luminex^®^ FLEXMAP 3D platform (Luminex Corporation, Austin, TX, United States) was used for data acquisition after adjusting the microparticle region for each analyte. After setting the instrument flow rate to 60 μL/min, the acquired median fluorescence intensity (MFI) values from the standards were used to create standard curves for each individual analyte, which were then used to calculate the analyte concentrations in each sample.

### 2.9 Statistical analysis

Data analysis was carried out using the Data Analysis ToolPak in Microsoft^®^ Excel version 16.34 (Microsoft^®^ Office 365, Redmond, DC, United States), and the figures were created using GraphPad Prism Version 9.4.0 (GraphPad Software Inc., San Diego, CA, United States) and collated using adobe illustrator version 27.4 (Adobe Inc. San Jose, CA, United States). Statistical significance was evaluated using Welch’s t-test. Statistical significance was considered when *p*-value is less than 0.05.

## 3 Results

### 3.1 Glass improved keratinocytes proliferation throughout the first 72 h

**FIGURE 1:**
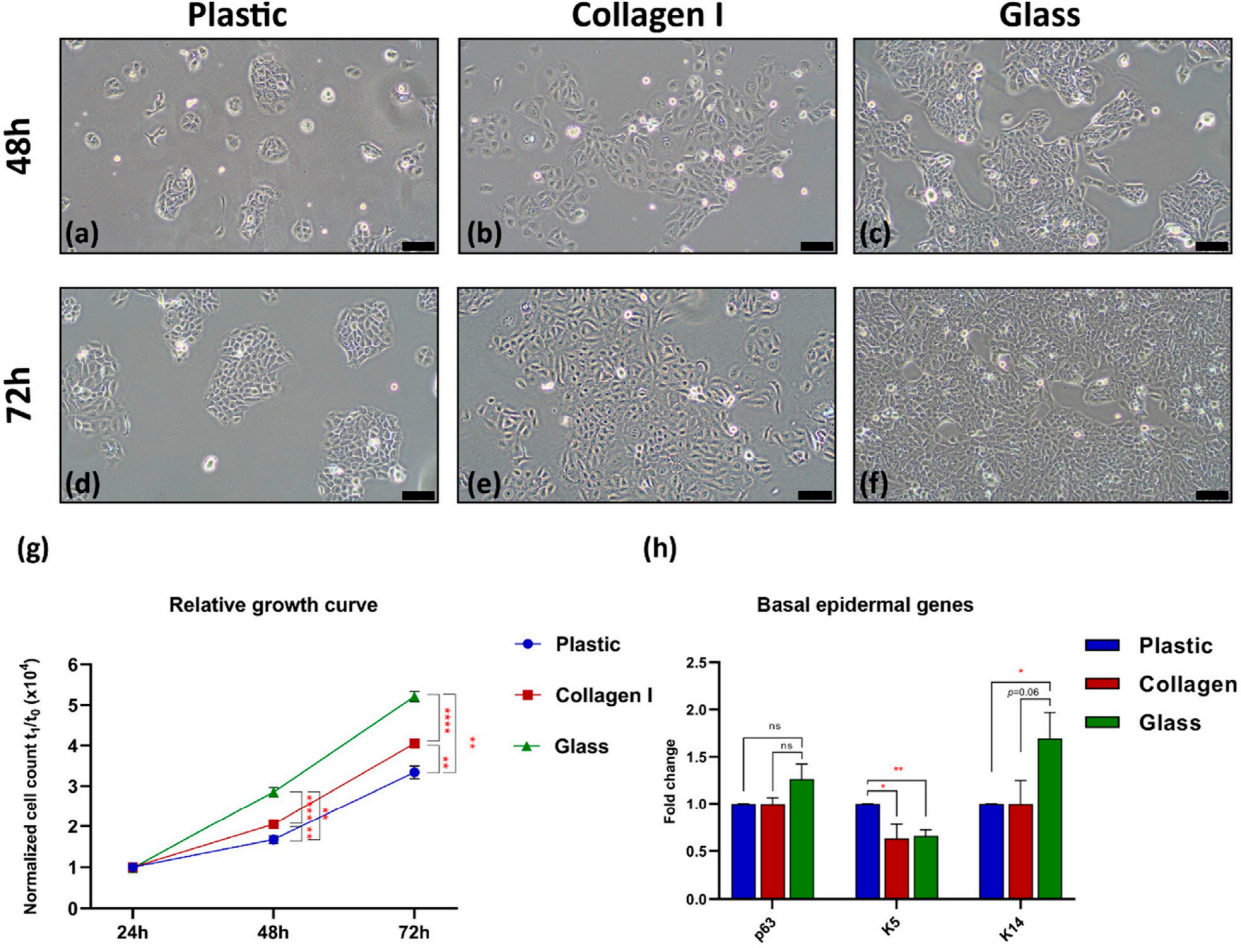
Live images showed HaCaT’s polygonal ‘cobblestone’ shape and colony formatiom **(a–c)** at 48 h and **(d–f)** at 72 h; scale bar = 100 µm. Images are representatives of four independent experiments. **(g)** HaCaT cultured on three substrates maintaining an exponential growth curve over 24, 48 and 72 h. Cells cultured on glass showed superior proliferation rate. **(h)** Gene expression of basal epidermal genes after 10 days in culture on the three substrates. p63 showed no significant changes between the three groups, K5 showed slight downregulation at FC of 0.6 and 0.7 in cells grown on collagen I and glass respectively, K14 showed upregulation in cells grown on glass by 1.7 FC compared to plastic. Gene expression data is shown as the mean of fold change of four independent experiments and the standard error of mean, expression was normalized to the plastic control FC of 1. *Statistically significant *p < 0.05, **p < 0.01, ***p < 0.001, & ****p < 0.0001 vs. the control.

Live imaging showed that the glass substrate supported expanding HaCaT colonies throughout the initial 48–72 h of seeding compared to the smaller dispersed patches observed in colonies on polystyrene (plastic) and collagen I substrates (Figures 1A–F). The proliferation rate of HaCaT was recorded by calculating the average change in cell count at 72 h normalized to its corresponding cell count recorded after 24 h of culture. Keratinocytes in all groups maintained an exponential growth curve during the recorded time points. However, cells grown on collagen I and glass showed significantly enhanced proliferation compared to the cells grown on plastic (control group) at 48 h (*p*-value = 0.007 and 1.4584E-06, respectively) and at 72 h (*p*-value = 0.003 and 8.78089E-08, respectively). At 48 h cell count in collagen I group increased by 110% and by 310% at 72 h compared to its initial count at 24 h (*p*-value = 2.39942E-08 and 1.40952E-07, respectively). In the glass group cell count increased by 180% at 48 h (*p*-value = 3.27252E-09) and by 430% at 72 h (*p*-value = 2.99342E-13) compared to its initial count at 24 h. Furthermore, keratinocytes cultured on glass even showed superior proliferation rate to those grown on collagen I by 73% at 48 h and by 128% at 72 h (*p*-value = 4.95103E-05 and 2.43764E-07, respectively) (Figure 1G).

To further explore the effect of glass on keratinocytes, the expression levels of epidermal lineage specific genes in the expanded keratinocytes were quantified using quantitative real-time PCR. Gene expression analysis indicated minor changes in the studied basal epidermal markers. The principal epidermal transcription factor, p63, showed no significant changes between the three groups. The basal-epidermal marker, keratin 14 (K14) showed clear upregulating effect of glass on cells; where K14 expression increased by 1.7 at fold change (FC) compared to plastic (p-value = 0.042). Keratin 5 (K5) showed downregulation of 0.6 and 0.7 FC in cells grown on collagen I and glass respectively compared to plastic (p-value = 0.046 and 0.006 respectively) (Figure 1H).

### 3.2 Primary keratinocytes cultured on glass exhibited global DNA demethylation

**FIGURE 2:**
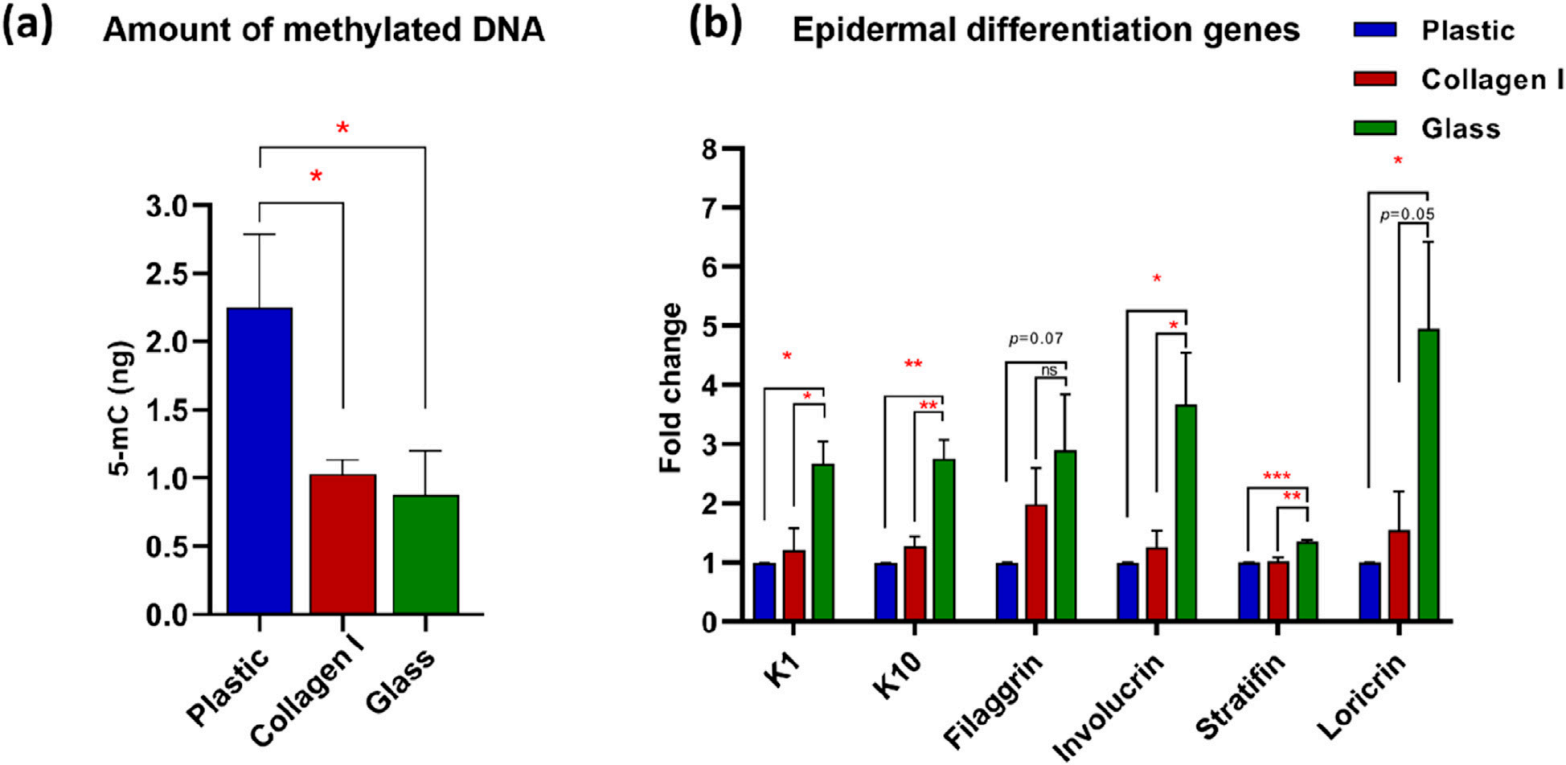
**(a)** Global DNA methylation level. Quantification of 5-mC content of DNA samples from keratinocytes cultured on studied substrates. Glass and collagen I decreased the methylation level significantly to almost 50% (0.9 ± 0.3 ng and 1 ± 0.1 ng, respectively) of the plastic control (2.3 ± 0.5 ng). **(b)** Gene expression analysis of epidermal differentiation markers shows upregulation in cells grown on glass of K1, K10, involucrin, stratifin and loricrin; whereas filaggrin shows an upregulation trend in glass compared to plastic at FC of 2.9. Gene expression data was presented as the mean of fold change of four independent experiments and the standard error of mean, expression was normalized to the plastic control FC of 1. *Statistically significant *p < 0.05, **p < 0.01 & ***p < 0.001 vs. the control.

To validate the effect of the studied surfaces on cell fate altering events, methylated DNA in the harvested keratinocytes at 10 days of culture on the investigated substrates was evaluated. Our analysis showed significant decrease in DNA methylation level in cells cultured on glass (0.9 ± 0.3 ng) in comparison to plastic (2.3 ± 0.5 ng) *(p*-value = 0.046). A similar effect was observed in keratinocytes cultured on collagen I (1 ± 0.1 ng) compared to those cultured on plastic (2.3 ± 0.5 ng) *(p*-value = 0.045) (Figure 2A).

### 3.3 Molecular characterization revealed enhanced epidermal differentiation of HaCaT cultured on glass

To investigate the effect of these surfaces on the adhered keratinocytes at the molecular level, a panel of epidermal differentiation markers, specific to suprabasal keratinocytes (i.e., stratum granulosum and corneum) were screened using quantitative real-time PCR. After 10 days in culture, cells on glass exhibited consistent upregulation across the studied suprabasal markers at the early and late end of the epidermal differentiation spectrum. The stratum granulosum-specific markers keratin 1 (K1), keratin 10 (K10), and filaggrin showed upregulation in the glass group with FC 2.7, 2.8 and 2.9 respectively. The upregulation of K1 was statistically significant in glass group compared to plastic and collagen I (*p*-value = 0.01 and 0.02 respectively). Similarly, K10 was significantly upregulated in glass group compared to plastic and collagen I (both at *p*-value = 0.006). Filaggrin on the other hand, exhibited a statistical trend with the glass group at FC of 2.9 compared to plastic *(p*-value = 0.07). However, keratinocytes cultured on collagen I were at FC of two for filaggrin expression compared to plastic; albeit without reaching statistical significance *(p*-value = 0.1). The expression of the latest epidermal differentiation genes represented by the stratum corneum-specific genes stratifin, involucrin, loricrin showed consistent upregulation in cells cultivated on glass at FC of 1.4, 3.7 and 4.5 respectively. Despite the modest FC upregulation of stratifin, the glass group expression presented a strong statistical significance compared to collagen I and plastic *(p*-value = 0.003 and 0.0008 respectively). The noted upregulation of involucrin showed statistical significance in glass group compared to plastic and collagen I (both at *p*-value = 0.03). Similarly, the enhancing effect of glass on loricrin expression was statistically significant but only when compared to plastic *(p*-value = 0.037) (Figure 2B).

### 3.4 Functional characterization revealed temporal reduction in thrombospondin-1 released by HaCaT cultured on glass with no change in the gene expression of the studied ECM markers

**FIGURE 3:**
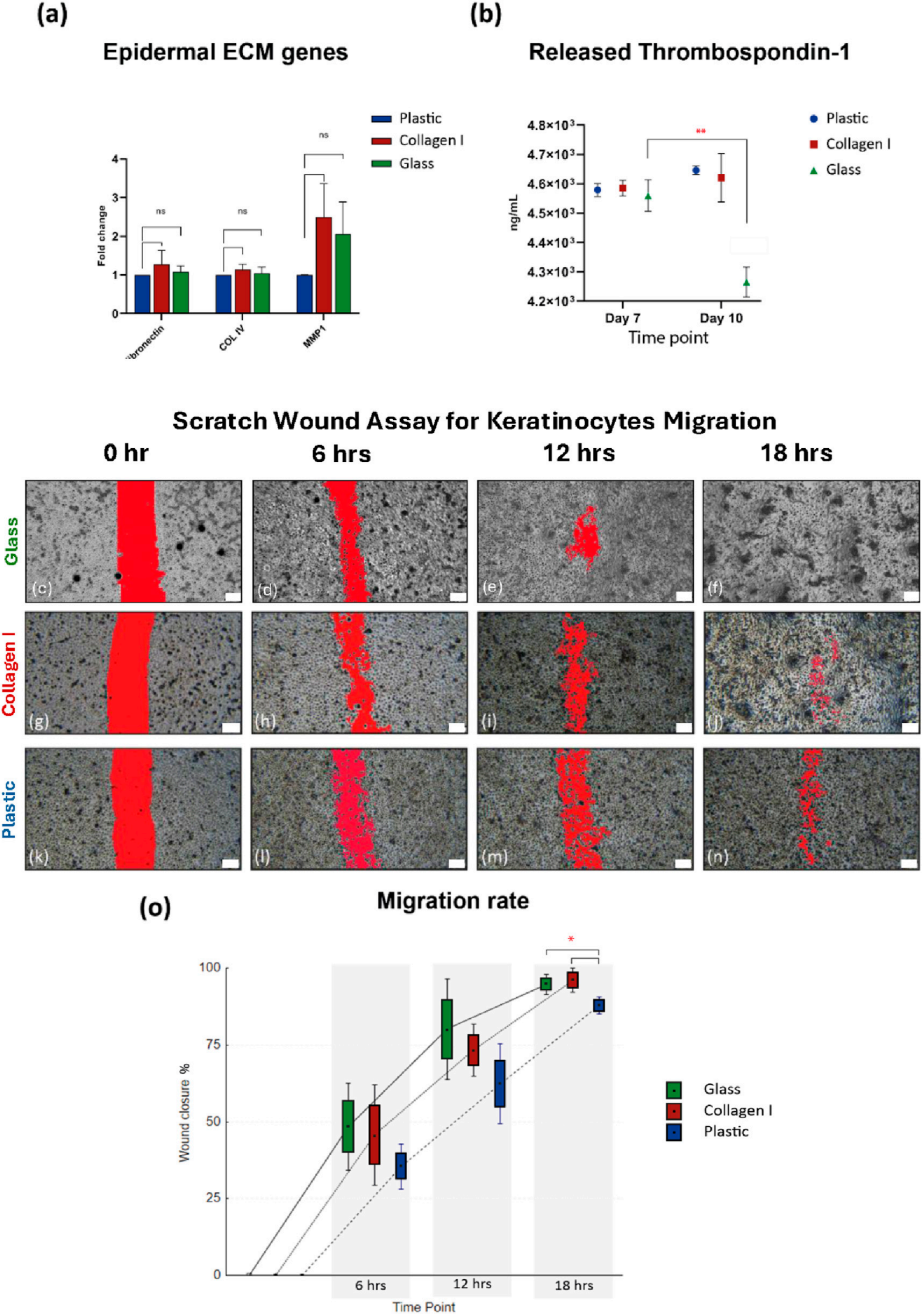
**(a)** Gene expression of ECM markers, shows comparable expression of fibronectin, Col IV and MMP1 after 10 days of culture on the three substrates. Gene expression data is shown as the mean of fold change of four independent experiments and the standard error of mean. **(b)** Comparable amounts of thrombospondin-1 released by keratinocytes cultured on all three substrates at ∼ 4.6 × 10^3^ ng/mL at day 7, then at day 10 thrombospondin-1 released by keratinocytes cultured on glass declined to 4.2 × 10^3^ ng/mL compared to day 7 (*p*-value = 0.008). **(c–f)** SWA images from glass group, showing faster cell migration in the glass group at 6, 12 & 18 h **(g–j)** SWA images from collagen I group, **(k–n)** SWA images from plastic group. Scale bars = 200 µm. **(o)** After 6 h the scratch wounds in glass and collagen I groups showing 48.4% and 45.8% healing respectively compared to 35.5% in the plastic group, at 12 h healing% shows 80.2%, 73.4% in glass and collagen I respectively compared to plastic 62.4%, at 18 h the scratch wounds almost entirely healed in the glass and collagen I groups with 94.8% and 96% respectively compared to plastic 87.9% closure (*p*-value = 0.02). SWA data is presented as Box: mean ± SE; Whisker: mean ± SD from three independent biological replicates. *Statistically significant *p < 0.05 & **p < 0.01 vs. the control.

Quantitative real-time PCR analysis showed no significant difference in the studied ECM markers at the gene expression level. The expression of fibronectin, Col IV and matrix metalloprotease (MMP) one were comparable in HaCaT after 10 days of culture on plastic, collagen I and glass. The expression level of MMP1 showed a slight elevation in HaCaT cultured on collagen I and glass at FC of 2.5 and 2, in comparison to plastic. However, neither of these markers reached statistical significance *(p*-value = 0.09 and 0.1 respectively) (Figure 3A).

Further mechanistic changes were investigated by characterizing the amount of thrombospondin-1 secreted in the culture supernatant, as a major adhesive glycoprotein and a regulator for cell-to-epidermal ECM interaction. The level of THBS1 in the supernatant of HaCaT cultured on glass, collagen I and plastic at day 7 were 4.56, 4.59 and 4.58 × 10^3^ ng/mL, respectively. At day 10, THBS1 level for collagen I and plastic groups remained around 4.6 × 10^3^ ng/mL. However, THBS1 concentration declined significantly in the glass group (4.2 × 10^3^ ng/mL) at day 10, compared to plastic and collagen I at the same time point (*p*-value = 0.006 and 0.01, respectively), as well as compared to the earlier glass recorded THBS1 concentration at day 7 (*p*-value = 0.008) (Figure 3B).

### 3.5 Scratch wound assay revealed that glass enhanced the migration of primary keratinocytes

To further validate the downregulating effect of THBS1 by glass on keratinocytes adhesion, cell migration was studied using SWA. Keratinocytes cultured on glass and collagen I migrated at a faster rate across the scratch over the course of 24 h, compared to those cultured on plastic. The scratch wound healed faster in the glass group signified by a larger number of migrated cells observed in the scratch area at each of the studied time-points, followed by cells grown on collagen I then plastic (Figures 3C–N). After the first 6 h, the scratch wounds in the glass and collagen I groups were 48.4% and 45.8% healed respectively compared to a mere 35.5% in the plastic group. A similar trend was noted at 12 h post-wounding as healing was at 80.2, 73.4% and 62.4% in glass, collagen I and plastic groups respectively. Interestingly, after 18 h the scratch wounds almost entirely healed in the glass and collagen I groups with 94.8% and 96% wound closure whereas the plastic group was delayed at 87.9% closure (*p*-value = 0.02) (Figure 3O). SWA data showed initial signs that glass and collagen I promoted the migration potential of primary human keratinocytes with a tendency of advantage for glass at the earlier time points.

### 3.6 Multi-analyte analysis of the secretome of human keratinocytes reveals temporal and substrate-dependent changes

**FIGURE 4:**
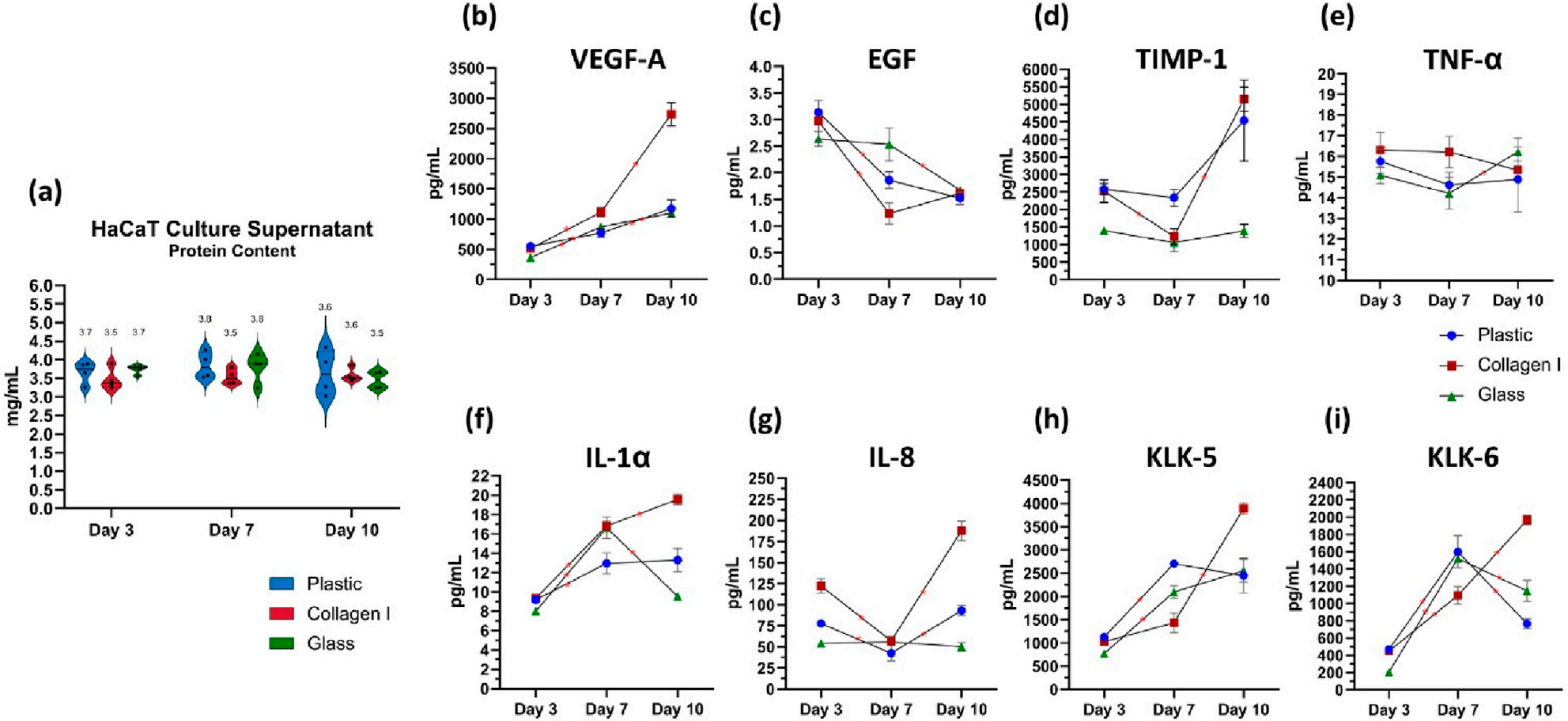
**(a)** Quantification of total protein content in the culture supernatant of keratinocytes. The data are presented as a violin plot, showing all individual data points. The borders of the violin represent the distribution, while the solid lines indicate the median of each dataset. The dotted lines denote the upper and lower quartiles, and the mean is shown as a separate marker. **(b–i)** Luminex^®^ multiplex immunoassays quantifying the levels of analytes secreted by cultured keratinocytes on days 3, 7 and 10. The analytes measured are **(b)** VEGF-A, **(c)** EGF, **(d)** TIMP-1, **(e)** TNF-α, **(f)** IL-1α, **(g)** IL-8, **(H)** KLK-5, and **(i)** KLK-6. Data from four independent experimental replicates are presented as superimposed symbols representing the mean ± standard error of the mean, with connecting lines illustrating trends. The data are normalized to the total protein content of each corresponding sample. Statistical significance was considered when p < 0.05 using Welch’s t-test. Statistical differences between each surface are detailed in Table 2. * Signifies statistical significance in the temporal differences between time points within each group.

Total protein content secreted by keratinocytes in the culture supernatant was quantified. The levels were comparable across the different culture substrates, with a mean ± SEM of 3.6 ± 0.1, 3.7 ± 0.1, and 3.6 ± 0.1 mg/mL on days 3, 7, and 10, respectively. No significant differences were observed between the culture groups or in the temporal trends across the time points (Figure 4A).

Keratinocytes seeded on glass recorded the lowest levels of Vascular Endothelial Growth Factor A (VEGF-A) release on day 3, with 363 ± 5 pg/mL, compared to 551 ± 32 pg/mL in the plastic group (*p* = 0.01) and 512 ± 4 pg/mL in the collagen I group (*p* = 0.00001). Over time, cells cultured on both plastic and glass showed a steady, albeit slight, increase in VEGF-A secretion, with similar levels recorded on day 7 at 767 ± 70 pg/mL and 870 ± 27 pg/mL, respectively (*p* = 0.1). This trend remained consistent on day 10, with VEGF-A levels increasing to 1,175 ± 144 pg/mL on plastic and 1,099 ± 69 pg/mL on glass (*p* = 0.3), with no significant divergence in their trajectories. Keratinocytes cultured on collagen I on the other hand, released VEGF-A in the highest amounts, with a consistent and significant increase over time. VEGF-A levels doubled from 512 ± 4 pg/mL on day 3–1,114 ± 72 pg/mL on day 7 (*p* = 0.007), continuing to rise steeply until day 10, reaching 2,736 ± 190 pg/mL (*p* = 0.0007). This value was significantly higher than those observed in the plastic group (1,175 ± 144 pg/mL, *p* = 0.0006) and the glass group (1,099 ± 69 pg/mL, *p* = 0.001) (Figure 4B). The release of epidermal growth factor (EGF) was consistently low across all three surfaces on day 3, averaging 2.9 ± 0.1 pg/mL. EGF levels subsequently declined on plastic (1.9 ± 0.2 pg/mL) and collagen I (1.2 ± 0.2 pg/mL) by day 7, compared to their respective day 3 baselines (*p* = 0.001 and 0.01, respectively). In contrast, EGF release was significantly more prolonged on glass surfaces until day 7; the decline in EGF on glass only occurred by day 10, plummeting to 1.7 ± 0.1 pg/mL (*p* = 0.02 and 0.03 compared to its day 3 baseline and day 7 level, respectively) (Figure 4C).

The detected amount of TIMP-1 was significantly lower on glass at day 3 compared to cells on plastic and collagen (*p* = 0.002 & 0.02 respectively). Additionally, TIMP-1 levels remained consistently lower in the glass group compared to plastic at day 7 (*p* = 0.01) and compared to both plastic and collagen I at day 10 (*p* = 0.036 and 0.001, respectively) (Figure 4D). TNF-α released by keratinocytes cultured on plastic and collagen I remained largely unchanged between the studied time points. However, when cultured on glass, there was a slight increase in TNF-α release from 14 ± 0.8 pg/mL on day 7–16 ± 0.7 pg/mL on day 10 (*p* = 0.049). Despite this increase, there was no statistically significant difference in TNF-α release among cells cultured on the three different surfaces (Figure 4E).

IL-1α levels in keratinocytes cultured on plastic exhibited an initial slight increase from 9.2 ± 0.8 pg/mL on day 3–13 ± 1.1 pg/mL on day 7 (*p* = 0.02), remaining steady at 13.3 ± 1.2 pg/mL by day 10 (*p* = 0.4). In contrast, keratinocytes cultured on glass showed a steep 100% increase of IL-1α from 8.0 ± 0.1 pg/mL on day 3–16.6 ± 1.1 pg/mL on day 7 (*p* = 0.002). Meanwhile, keratinocytes on glass released the lowest amount of IL-1α on day 10 (9.5 ± 0.4 pg/mL), compared to those in the plastic group (13.3 ± 1.2 pg/mL) (*p* = 0.02) and collagen I group (19.6 ± 0.6 pg/mL) (*p* = 0.00001). Notably, the collagen I group exhibited the steepest rise in IL-1α, increasing from 9.3 ± 0.4 pg/mL on day 3–16.8 ± 0.5 pg/mL by day 7 (*p* = 0.00009), and peaking at 19.6 ± 0.6 pg/mL by day 10 (*p* = 0.006). (Figure 4F). The level of IL-8 released on day 3 was highest in the collagen I group (122 ± 9 pg/mL) compared to the plastic group (78 ± 4 pg/mL) (*p* = 0.004). Cells cultured on glass exhibited the lowest IL-8 secretion on day 3 (54 ± 1 pg/mL) relative to both plastic and collagen I (*p* = 0.01 and 0.002, respectively). By day 10, cells on collagen I recorded the highest IL-8 levels (188 ± 11 pg/mL), significantly higher than the plastic group (93 ± 6 pg/mL) (*p* = 0.0006). In contrast, the attenuated IL-8 secretion in the glass group persisted throughout, with levels of 56 ± 5 pg/mL on day 7 and 50 ± 5 pg/mL on day 10. No significant temporal changes in IL-8 secretion were observed in cells cultured on glass (Figure 4G).

On day 3, the amount of released Kallikrein-5 was lowest in keratinocytes cultured on glass (772 ± 20 pg/mL) compared to those on plastic (1,127 ± 76 pg/mL) and collagen I (1,026 ± 70 pg/mL) (*p* = 0.008 and 0.03, respectively). By day 7, KLK-5 levels had increased in both plastic (2,703 ± 35 pg/mL) and glass (2,096 ± 135 pg/mL) compared to their respective levels on day 3 (*p* = 0.00002 and 0.001, respectively). On day 10, a notable increase in KLK-5 was observed in cells cultured on collagen I, with levels rising from 1,430 ± 211 pg/mL on day 7–3,890 ± 130 pg/mL (*p* = 0.00009). In contrast, KLK-5 levels remained stable in both plastic and glass groups compared to day 7 (*p* = 0.3 and 0.1, respectively) (Figure 4H). The amount of kallikrein-6 released by keratinocytes on day 3, in response to different seeding substrates, followed a pattern similar to that of KLK-5. Keratinocytes seeded on glass released significantly lower levels of KLK-6 (205 ± 15 pg/mL) compared to those on plastic (470 ± 21 pg/mL) and collagen I (449 ± 40 pg/mL) (*p* = 0.005 and 0.003, respectively). By day 7, KLK-6 levels increased in all groups, reaching 1,598 ± 188 pg/mL on plastic, 1,521 ± 28 pg/mL on glass, and 1,094 ± 103 pg/mL on collagen I (*p* = 0.004, 0.0000003, and 0.03, respectively, compared to day 3). Interestingly, cells cultured on collagen I continued to increase their KLK-6 secretion, peaking at 1967 ± 54 pg/mL on day 10, similar to the trend observed for KLK-5. In contrast, KLK-6 levels dropped sharply in the plastic (768 ± 59 pg/mL) and glass (1,146 ± 123 pg/mL) groups (*p* = 0.00001 and 0.002, respectively) (Figure 4I). Statistical differences between each surface are listed in Table 2.

**TABLE 2:** Statistical differences between the amounts of analytes detected in the culture supernatant of each group.

	Day 3			Day 7			Day 10		
	Plast vs. Col	Plast vs. glass	Col vs. glass	Plast vs. Col	Plast vs. glass	Col vs. glass	Plast vs. Col	Plast vs. glass	Col vs. glass
VEGF-A	0.2	0.01	0.000010	0.01	0.1	0.03	0.0006	0.3	0.0008
EGF	0.4	0.065	0.24	0.02	0.060	0.008	0.3	0.2	0.3
TIMP-1	0.4	0.002	0.02	0.01	0.01	0.3	0.3	0.036	0.001
TNF-a	0.3	0.2	0.1	0.078	0.3	0.057	0.4	0.2	0.2
IL-1a	0.4	0.1	0.01	0.02	0.03	0.4	0.004	0.02	0.000006
IL-8	0.004	0.01	0.002	0.1	0.1	0.4	0.0006	0.0009	0.0002
KLK-5	0.2	0.008	0.03	0.004	0.009	0.02	0.02	0.4	0.007
KLK-6	0.3	0.005	0.003	0.039	0.4	0.06	0.00001	0.02	0.002

## 4 Discussion

Wound healing is a coordinated, progressive, multi-stage process that involves blood coagulation, inflammation, re-epithelialization by migrating keratinocytes, granulation tissue formation, angiogenesis, and, ultimately, tissue remodelling (Koivisto et al., 2014). These stages are activated in successive synergy to restore the barrier function and integrity of the epithelium. Proliferation, differentiation and migration are defining features in keratinocytes wound repair potential and reepithelialisation ability. During the initial stages of keratinocytes expansion, *in-vitro*, cells strive to switch from the expanding mode to reach a balanced mode between dividing and differentiating keratinocytes to achieve the population asymmetry mimicking basal keratinocytes behaviour *in-vivo* necessary to sustain epidermal homeostasis (Roshan et al., 2016). Our initial observation showed that glass substrates supported more spread-out clusters of expanding keratinocytes colonies which sustained the log phase in the keratinocytes growth curve throughout the first 72 h of cultivation, compared to the smaller patches observed in keratinocytes colonies on polystyrene (plastic) substrate. This observation comes in agreement with earlier report showing a positive correlation between the substrate stiffness and the spread of keratinocytes. Keratinocytes cultured on stiff polyacrylamide gels embedded with fluorescent microspheres exhibited a notably increased degree of cell spreading (Zarkoob et al., 2015).

The effect of substrate on cultured keratinocytes can be prompted by the global DNA demethylation shown at day 10. Consistent with this observation, DNA methyltransferase one is a key enzyme expressed in epidermal progenitor cells to maintain DNA methylation patterns after cellular replication. This enzyme was found to be progressively downregulated throughout epidermal differentiation (Sen et al., 2010). The effect of glass on decreasing global DNA methylation was previously shown in stem cell model cultured on glass in three dimensions. The reduction in DNA methylation level was accompanied by upregulation of the osteogenic differentiation regulators Runx-2 and osterix alongside ECM gene (collagen I). Furthermore, the osteogenesis and angiogenesis-related growth factors, transforming growth factor beta (TGFß) and vascular endothelial growth factor (VEGF), were also upregulated (Alghfeli et al., 2021; Alghfeli et al., 2022).

In our screening of lineage specific genes, a master epidermal regulator and a proliferation marker, keratin 14, was the only upregulated basal epidermal marker in cells on glass at day 10. This upregulation supports our earlier observation about glass enhancing the proliferative capacity of keratinocytes in monolayer culture. The studied panel of genes related to stratified epithelial differentiation, Keratin 1 and 10, filaggrin, stratifin, involucrin and loricrin were uniformly upregulated in response to cell contact with glass. These markers are essential factors in epidermal stratification that contribute to formation of the cutaneous barrier (Truong et al., 2006). This pattern of gene expression is consistent with the reported effect of incorporating bioactive glass extracts with the keratinocytes culture medium at various dilutions. This study reported enhanced epidermal differentiation where K10 and involucrin were significantly upregulated by the glass extracts in a concentration dependent manner. K14 was also upregulated but in a bioactive glass extracts concentration independent manner compared to keratinocytes cultured in medium without glass extracts (Tang et al., 2021). The correlation between DNA demethylation and enhanced epidermal differentiation was recapitulated via the treatment of human keratinocytes with the DNA demethylating agent 5-azacytidine over a period of 24 h. The treated cells strikingly morphed to resemble differentiating keratinocytes characterized by pronounced enlargement and flattening of the cells within 5–7 days following removal of 5-azacytidine. The effect was confirmed by the upregulation of the epidermal differentiation gene complex including suprabasal and stratum corneum-associated keratins typically induced during the epidermal differentiation (Okada et al., 1984; Kohler and Rodriguez-Paredes, 2020).

Collagen IV and fibronectin are essential structural proteins produced by keratinocytes and function as cell anchors to the cutaneous ECM. For successful wound healing to take place, keratinocytes must first be able to detach from the underlying basal lamina, then MMPs facilitate keratinocyte migration through the newly synthesized ECM of the wound. MMP1 is expressed abundantly at the wound edges and involved in sustaining keratinocyte migration and the formation of granulation tissue during wound healing (Pastar et al., 2014; Evan et al., 2018). Functional characterization revealed comparable expression of the ECM genes: collagen IV, fibronectin and MMP1, between keratinocytes cultured on all three substrates. The evaluation of thrombospondin-1 as a secreted protein showed inhibition at day 10 in keratinocytes cultured on glass. Thrombospondin-1 is an adhesive matricellular glycoprotein that mediate cell-matrix interactions and cytokine activation and play significant roles in tissue remodelling and fibrosis (Murphy-Ullrich, 2019). In the context of cutaneous homeostasis, thrombospondin-1 inhibition occurs when keratinocytes located in the basal cell layer commence differentiation. The cells start to lose their attachment to the basement membrane as a result of thrombospondin-1 inhibition and migrate from the basal cell layer and occupy the suprabasal layers along the stratified epithelium (Varani et al., 1988). Overexpression of thrombospondin-1 delayed the healing in mouse full-thickness skin wounds, diminished formation of granulation tissue and severely impaired wound angiogenesis (Koivisto et al., 2014). The role of thrombospondin-1 as endogenous inhibitor of angiogenesis has been established in hypoxic skin. Conditioned media from human microvascular endothelial cells cultured in hypoxic conditions showed higher expression of thrombospondin-1 and actively impaired endothelial cell proliferation. The endothelial cell anti-proliferative effect was mitigated by thrombospondin-1 depletion from the media and reactivated by addition of exogenous thrombospondin in a dose-dependent manner (Morgan-Rowe et al., 2011). *In-vitro* and *in-vivo* studies have recenlty demonstrated the advantageous effect of different formulations of bioactive glass on the angiogenic and regenerative properties of bone-marrow mesenchymal stem cells and proven to be a glass composition-dependent effect (Qazi et al., 2018; Westhauser et al., 2019).

Vascular Endothelial Growth Factor A (VEGF-A), the most studied and predominant isoform of VEGF, plays a key role in promoting angiogenesis and endothelial cell proliferation. VEGF-A is also involved in increasing vascular permeability (Bae et al., 2015). In the skin, this growth factor is released by keratinocytes and fibroblasts following injury, playing a key role in the early stages of angiogenesis through promoting endothelial cell migration and proliferation. During the initial phase of wound healing, platelets also release VEGF in response to hypoxia. Acting in a paracrine manner, VEGF targets both endothelial and immune cells, facilitating reepithelialization, initiating new blood vessel formation, and restoring oxygen supply to the damaged tissue (Johnson and Wilgus, 2014; Pastar et al., 2014; Bae et al., 2015). VEGF-A quantification in the culture supernatant showed an increase across all three substrates; however, the collagen I matrix had the most pronounced effect on VEGF-A production. Keratinocytes cultured on glass and plastic exhibited a steady, marginal increase in VEGF-A release. In contrast, cells grown on collagen I showed significant surges in VEGF-A production throughout the cultivation period, culminating in a dramatic threefold increase compared to keratinocytes cultured on glass and plastic by day 10. This pronounced effect of collagen I on VEGF-A production, an angiogenic marker, among the tested analytes in the keratinocyte secretome aligns with the well-established reciprocal modulatory relationship between collagen I, the most abundant structural protein in the extracellular matrix (ECM), and VEGF-A. VEGF has been linked to dermal repair by influencing collagen production and organization in wound healing. Studies show a strong correlation between VEGF levels and the formation of granulation tissue, with VEGF also impacting wound breaking strength, potentially through changes in collagen synthesis or its structural arrangement (Johnson and Wilgus, 2014). A study using a murine model of scarless fetal repair and adult wound healing found that VEGF levels are directly associated with the extent of scar tissue formation. Systemic administration of neutralizing VEGF antibodies reduced scar size and restored normal collagen fibril structure in adult incisional wounds. Conversely, injecting recombinant VEGF into embryonic day 15 fetal wounds, which typically heal without scarring, led to the development of large scars (Wilgus et al., 2008). Conversly, collagen I plays a regulatory role in VEGF synthesis and activity, particularly in processes involving tissue repair, fibrosis, and angiogenesis. This effect was reported when sub-confluent human umbilical vein endothelial cells (HUVECs) were cultured on polyacrylamide (PA) gels coated with type I collagen or fibronectin, with varying stiffness. Matrix stiffness enhanced the VEGF-induced VEGFR-2 response, with a more pronounced effect in cells cultured on collagen-coated PA gels. The study demonstrated that ECM stiffness promotes VEGFR-2 internalization and downstream VEGF-stimulated signaling, leading to increased proliferation (LaValley et al., 2017).

Our previous research revealed EGF and IL-1α to be the only two proteins that were significantly overproduced by keratinocytes when comparing the proteome signatures of human keratinocytes and adipose-derived mesenchymal stem cells (Shahin et al., 2023). Epidermal Growth Factor (EGF) is a critical regulator of epidermal homeostasis. Endogenous to human keratinocytes, EGF plays a pivotal role in maintaining barrier function, promoting terminal differentiation, regulating cell adhesion, and controlling protease secretion. Beyond its role in epidermal homeostasis, EGF functions as a signaling factor that supports effective wound closure. EGF ensures efficient skin repair by stimulating keratinocyte and fibroblast proliferation, modulating angiogenesis and collagen synthesis, and promoting epithelialization (Barrientos et al., 2008). The release of EGF from HaCaT cells cultured on different substrates was quantified. Glass substrates sustained EGF release for a prolonged period of 7 days, while plastic and collagen I substrates exhibited a noticeable decline in EGF levels. This finding suggests that 7-day cultures on glass may be optimal for obtaining keratinocytes that are primed for transplantation. EGF has been extensively studied for its potential as a topical treatment. A study demonstrated that a combination of EGF and tocotrienol-rich fraction (TRF) accelerated wound healing in rats with burn injuries (Guo et al., 2020). Clinically, EGF has proven effective in treating diabetic foot ulcers. A meta-analysis revealed that patients receiving EGF experienced significantly higher rates of complete wound healing compared to placebo, regardless of the administration route (Bui et al., 2019). The pivotal role of EGF in promoting cutaneous wound healing is underscored by its ability to stimulate keratinocyte migration through the regulation of α2 integrin expression (Chen et al., 1993). This mechanism, coupled with its established role in maintaining epidermal homeostasis, highlights the significance of EGF as a valuable therapeutic agent for wound repair. To maximize the therapeutic potential of EGF, it is essential to employ cell culture systems that sustain its release into the wound bed. Our 7-day on-glass culture approach can ensure that keratinocytes transplanted into wounds are primed with adequate levels of EGF, thereby enhancing their capacity for tissue regeneration and accelerating wound closure.

Epidermal keratinocytes, similar to all epithelial cells with a barrier function, are rich in IL-1α in the physiological state. As a major immune modulatory cytokine, IL-1α facilitates wound healing by inducing fibroblast and keratinocyte proliferation, the regulation of ECM proteins, fibroblast chemotaxis, and by exerting immune modulatory functions. However, the role of IL-1α in wound healing is nuanced, requiring a careful balance between its synthesis and degradation (Macleod et al., 2021). Upon skin injury, trauma, or infection, IL-1α is promptly released, reaching its peak in the first 12–24 h initiating local inflammation and facilitating the recruitment of neutrophils to the wound site, thereby removing debris and preventing bacterial infection (Cavalli et al., 2021). As an integral signalling factor of the IL-1 family cytokines, IL-1α mobilizes locally located epidermal stem cells, as well as enhances the keratinocyte migration (El-Serafi et al., 2022). However, prolonged elevation of IL-1α levels in the wound environment demarcates persistent inflammation. For wound to heal successfully, it is essential that IL-1α reverts to normal levels once the proliferation stage of wound healing is complete (Macleod et al., 2021). The proteolytic regulation of IL-1α has been demonstrated in a coagulation system where thrombin cleaves the native form of IL-1α known as pro-IL-1α at a specific, conserved site, activating the cytokine. The functional importance of this cleavage was demonstrated in a mouse model. Mice with a mutation that prevented thrombin from cleaving IL-1α exhibited impaired wound healing and thrombopoiesis (Burzynski et al., 2019). Our temporal analysis of IL-1α levels in the culture supernatant revealed distinct patterns among the three substrates. Keratinocytes cultured on glass released lower levels of IL-1α than those on collagen I at day 3. However, by day 7, IL-1α levels in both groups had increased to similar levels. Intriguingly, this trend continued for the collagen I group, with IL-1α levels rising further by day 10. In contrast, IL-1α levels in the glass group dropped significantly during this period, approaching baseline levels. The plastic control had no discernible effect on IL-1α release by the cells throughout the studied time points. While an initial IL-1α response is crucial for initiating inflammation and wound healing, excessive or prolonged IL-1α production can be detrimental. The findings presented in this study support the use of glass as a substrate for short-term expansion of keratinocytes for potential clinical applications. This is due to the observed balanced IL-1α release on glass, which may be beneficial for optimizing the inflammatory response and promoting efficient wound healing.

IL-8 is a pro-inflammatory cytokine that plays a critical role in immune responses and skin disorders. It is the most commonly reported keratinocyte secretion in culture supernatant. Produced by epithelial cells in response to infections or injuries, IL-8 attracts neutrophils to initiate inflammation and promotes angiogenesis by stimulating dermal microvascular endothelial cells to release MMP2 and MMP9. IL-8 interacts with Toll-like receptors on keratinocytes to establish the inflammatory conditions needed to combat infection (El-Serafi et al., 2022). Traditionally, reduced IL-8 levels in the stratum corneum correlate with treatment efficacy in atopic dermatitis, making it a faithful biomarker for monitoring therapeutic effects in these patients (Murata et al., 2021). In our results, glass consistently maintained low and steady levels of IL-8 release compared to both plastic and collagen I throughout the 10-day cultivation period. This suggests that glass may promote a mitigated inflammatory state in keratinocytes, potentially offering advantages for regenerative transplantation applications. Tumor necrosis factor (TNF-α) is another pro-inflammatory cytokine released in significant amounts by keratinocytes immediately after injury, initiating the inflammatory response and contributing to epidermal repair (El-Serafi et al., 2022). Persistently elevated TNF-α levels indicate an unresolved inflammatory response, which can disrupt skin barrier function, degrade extracellular matrix components, and impair skin regeneration and repair. This elevation is commonly associated with conditions such as psoriasis, eczema, and wound healing, where inflammation alters keratinocyte proliferation, differentiation, and immune activity (Banno et al., 2004; Pastar et al., 2014). In our results, there was no substantial effect of the tested substrates on the secreted levels of TNF-α. However, the slight temporal increase in TNF-α observed on glass between days 7 and 10 provides further evidence supporting the 7-day recommendation for culturing keratinocytes on glass.

Human tissue kallikreins (KLKs) represent a family of 15 highly conserved serine proteases, with KLK-5 and KLK-6 playing key roles in the molecular mechanisms governing epidermal differentiation in human keratinocytes. These proteases are encoded by a contiguous cluster of genes located on chromosome 19q13.4 and are critical in maintaining skin homeostasis and barrier function. KLK-5 is involved in desquamation, facilitating the proteolytic degradation of corneodesmosomes (Jiang et al., 2011). Consequently, KLK-5 has been recognized as a reliable marker for monitoring epidermal differentiation (Dos Santos et al., 2019). In contrast, KLK-6 participates in skin remodeling and repair. Studies on KLK-6 knockout mice have shown a reduction in epidermal thickness and decreased keratinocyte proliferation. Additionally, activation of KLK-6 has been demonstrated to counteract the inhibitory effects of topical glucocorticoids on keratinocyte proliferation, underscoring its pivotal role in epidermal regeneration (Kishibe et al., 2016). In our secretome analysis, an early surge of released KLK-5 and KLK-6 was observed between days 3 and 7 in cells grown on plastic and glass. This surge was less pronounced in cells cultured on a collagen I matrix, where peak levels were noted at day 10. This suggests that glass accelerated epidermal differentiation by day 7, compared to the delayed yet significant effect observed with collagen I at day 10. This observation aligns with gene expression trends in epidermal differentiation markers, further supporting the pronounced effect of glass on both epidermal differentiation and proliferation.

During epidermal maturation, a proliferative basal layer of epidermal stem cells undergoes programmed differentiation and start to mature as they migrate upward within the skin, giving rise to multiple suprabasal layers (Pastar et al., 2014). Migration is a defining feature of differentiating keratinocytes and a decisive function in keratinocytes’ ability to maintain the epidermal physical barrier but also for its restoration upon injury. During epithelialization, keratinocytes at the wound edge loosen their adherence to the basal lamina and cell–cell and cell–substratum structures start dissociating. This release allows keratinocytes to close the defect in the epidermis by orchestrated effort between migrating and proliferating keratinocytes. Migrating keratinocytes travel from the wound edge over the open wound area, whereas keratinocytes behind the migrating tongue begin to proliferate (Blanpain and Fuchs, 2009; Pastar et al., 2014; Kohler and Rodriguez-Paredes, 2020). In this study, the effect of the experimental culture substrates on the migration rate of primary keratinocytes was recorded over the course of 24 h. We noticed that keratinocytes cultured on glass and collagen I showed enhanced migration compared to plastic. This data provides evidence that keratinocytes culture on glass accelerated differentiation and improved their migratory capacity. Consistent with previous findings, keratinocytes monolayer were cultured with various dilutions of ionic extracts from bioactive glass enhanced keratinocytes migration (Tang et al., 2021). Interestingly the authors noted keratinocytes migrated collectively as a sheet in a manner similar to that noted in our glass group. On the other hand, this study showed keratinocytes of the control group without glass extracts were scattered over the denuded area in what appears to be single-cell migration with no substantial cell-cell contact over the course of 24 h (Tang et al., 2021). MMPs are regulated by tissue inhibitors of metalloproteinases (TIMPs), a family of proteins (TIMP-1 to TIMP-4) with distinct protease inhibitory profile of each TIMP. Present in the ECM in soluble form, TIMP-1 particularly plays a key role in regulating tissue remodeling, wound healing, and maintaining the integrity of the extracellular matrix. TIMP-1 inhibits MMPs by forming reversible 1:1 stoichiometric complexes. Effective keratinocyte migration during wound healing relies on precise balance between MMP function and TIMP-1 thereby ensuring controlled matrix degradation, while imbalanced MMP/TIMP ratios are characteristic of chronic, non-healing wounds (Rohani and Parks, 2015; Cabral-Pacheco et al., 2020). Our TIMP-1 quantification in the culture supernatant revealed that keratinocytes on glass consistently released low levels of TIMP-1 across all time points. This finding aligns with the cell migration assay data, providing strong evidence that glass promotes enhanced keratinocyte migration.

These collective results from the on-glass approach demonstrated robust evidence of keratinocyte differentiation and enhanced migratory potential. We quantified this by measuring markers for epidermal differentiation and migration on the gene expression, proteomic and functional levels. Our findings suggest that this approach is a promising method for propagating keratinocytes in culture for a short duration with potential applications in regenerative transplantation therapies. Furthermore, culturing keratinocytes on glass may provide an *in vitro*, monolayer model for keratinocytes at a more advanced state of differentiation. This is particularly useful for testing dermatological products and locally acting medications, as current models rely on progenitor cells or keratinocytes at very early stages of differentiation. Culturing keratinocytes on glass can also aid in designing more physiologically relevant multilayer skin constructs for dermatological investigations.

## 5 Conclusion

This work shows evidence of the efficacy of glass as culture substrate to deliver readily initiated keratinocytes with efficacy for cell transplantation applications. From a clinical and practical standpoint, this approach provides a safe and simple mean to expand sheets of keratinocytes with potential usability in tissue engineering. This study has a limitation of the use of both primary keratinocytes and the normal keratinocyte cell line ‘HaCaT’. As some of the experiments require large numbers of cells, it was challenging to have sufficient primary cells, especially with the limited donated skin biopsies after the COVID-19 pandemic. The data from the 2 cell sources were complementary and no controversy was noticed during the preparation of this manuscript. Overall, our data showed that contact with glass improved keratinocytes differentiation, migration and functional characteristics pertinent to restoring barrier functions and wound angiogenesis; both are integral parts of epithelialization and granulation tissue formation. As a simple, ubiquitous and xeno-free material, glass appears to have brought moderate enhancements to expanding keratinocytes. In depth analysis of the effect of glass on keratinocytes, at the level of cell-cell and cell-matrix interactions in terms of cell adhesion, signalling cascades and spatial configuration would constitute interesting avenues for further elucidation. More importantly, pre-clinical efficacy and safety studies in cutaneous wound healing models are needed before implementing this glass approach in the workflow of cell-based regenerative therapies.

## Data Availability

The original contributions presented in the study are included in the article/supplementary material, further inquiries can be directed to the corresponding author.

## Abbreviations

K, Keratin; ECM, extracellular matrix; EMA, European Medicines Agency; ATMP, Advanced Therapy Medicinal Product; FBS, Foetal bovine serum; GAPDH, Glyceraldehyde 3-phosphate Dehydrogenase; Col IV, Collagen IV; MMP, Matrix metalloproteinase; THBS1, Thrombospondin-1; SWA, Scratch wound assay; TGFß, transforming growth factor beta; VEGF, vascular endothelial growth factor.
